# Attenuation of TRPV1 and TRPV4 Expression and Function in Mouse Inflammatory Pain Models Using Electroacupuncture

**DOI:** 10.1155/2012/636848

**Published:** 2012-11-13

**Authors:** Wei-Hsin Chen, Jason T. C. Tzen, Ching Liang Hsieh, Yung Hsiang Chen, Tzu-Jou Lin, Shih-Yin Chen, Yi-Wen Lin

**Affiliations:** ^1^Graduate Institute of Biotechnology, National Chung Hsing University, Taichung 40227, Taiwan; ^2^Graduate Institute of Acupuncture Science, China Medical University, 91 Hsueh-Shih Road, Taichung 40402, Taiwan; ^3^Acupuncture Research Center, China Medical University, Taichung 40402, Taiwan; ^4^Graduate Institute of Integrated Medicine, China Medical University, Taichung 40402, Taiwan; ^5^Graduate Institute of Basic Medical Science, China Medical University, Taichung 40402, Taiwan; ^6^School of Chinese Medicine, China Medical University, Taichung 40402, Taiwan

## Abstract

Although pain is a major human affliction, our understanding of pain mechanisms is limited. TRPV1 (transient receptor potential vanilloid subtype 1) and TRPV4 are two crucial receptors involved in inflammatory pain, but their roles in EA- (electroacupuncture-) mediated analgesia are unknown. We injected mice with carrageenan (carra) or a complete Freund's adjuvant (CFA) to model inflammatory pain and investigated the analgesic effect of EA using animal behavior tests, immunostaining, Western blotting, and a whole-cell recording technique. The inflammatory pain model mice developed both mechanical and thermal hyperalgesia. Notably, EA at the ST36 acupoint reversed these phenomena, indicating its curative effect in inflammatory pain. The protein levels of TRPV1 and TRPV4 in DRG (dorsal root ganglion) neurons were both increased at day 4 after the initiation of inflammatory pain and were attenuated by EA, as demonstrated by immunostaining and Western blot analysis. We verified DRG electrophysiological properties to confirm that EA ameliorated peripheral nerve hyperexcitation. Our results indicated that the AP (action potential) threshold, rise time, and fall time, and the percentage and amplitude of TRPV1 and TRPV4 were altered by EA, indicating that EA has an antinociceptive role in inflammatory pain. Our results demonstrate a novel role for EA in regulating TRPV1 and TRPV4 protein expression and nerve excitation in mouse inflammatory pain models.

## 1. Introduction

Pain, which affects more than 20% of the population worldwide, is a complicated therapeutic challenge with mechanisms that are not fully understood. Pain can be evoked by tissue damage, noxious environmental stimuli, hypoxia, acidosis, and inflammation [1, 2]. Tissue damage causes the injured regions to release inflammatory mediators such as bradykinin, prostaglandins, protons, and neurotransmitters, which activate nerve terminals to pain signal transduction [3].

The TRPV (transient receptor potential vanilloid) family is highly associated with nervous system functions such as pain, memory, and mechanical sensations [4]. The TRPV family includes six subtypes—TRPV1, TRPV2, TRPV3, TRPV4, TRPV5, and TRPV6—all of which are widely expressed in the mammalian central and peripheral nervous systems [5–8].

TRPV1 is usually considered to be involved in the perception of inflammatory and thermal pain, especially pain from heat above 43°C [4, 9]. TRPV1 is highly expressed in dorsal root ganglion (DRG) neurons, especially in C-fiber neurons, and activation of TRPV1 leads to sodium and calcium influx, causing cell depolarization [10, 11]. Depletion of TRPV1 results in decreased sensitivity to noxious heat and delays radial heat and hot-plate tests [12]. Luo et al. showed the change of the TRPV1 expression after CFA-induced inflammatory pain. TRPV1 protein was increased from day 1 to day 21 and reduced at day 28. Subcutaneous or intrathecal injection of TRPV1 antagonist capsazepine (CPZ) could reliably reduce CFA-induced thermal hyperalgesia [13, 14].

TRPV4 is highly associated with osmotic pressure and mechanical sensitivity and has been expressed in heterologous systems [15, 16]. Mice lacking TRPV4 have diminished regulation of serum osmolarity and are less sensitive to noxious stimuli [17, 18]. TRPV4 also participates in many different types of pain mediation, such as pain resulting from mechanical hyperalgesia and complications of vincristine chemotherapy, diabetes, alcoholism, and acquired immune deficiency syndrome therapy [19, 20]. Moreover, TRPV4 mutant mice showed normal behavior on thermal test after CFA injection and also participated in carrageenan- and inflammation-mediators-induced thermal and mechanical hyperalgesia [21–23]. 

Acupuncture is an ancient Chinese method for curing pain for more than 3000 years. However, the detailed mechanism of acupuncture effects remains an important unresolved issue [24]. Several studies have shown that injection with the local anesthetic procaine inhibits the analgesic effect of acupuncture [25–29].

Recently, several studies revealed that TPRV1 and TRPV4 are both involved in mechanical and thermal hyperalgesia [13, 14, 21–23], but few reports showed the relationship between acupuncture with TRPV1 and TRPV4. We have investigated whether TRPV1 and TRPV4 are key mediators for the effects of acupuncture therapy on inflammatory pain, as indicated by our previous research [30]. Our results demonstrate that electroacupuncture (EA) is effective in inducing analgesia in inflammation-induced hyperalgesia by downregulating TRPV1 and TRPV4 expression. 

## 2. Methods and Materials 

### 2.1. Animals and EA Pretreatment

Adult ICR (BioLASCO Taiwan Co., Ltd.) female mice aged 8 to 12 weeks were used in the experiment. The usage of these animals was approved by the Institute of Animal Care and Use Committee of China Medical University, Taiwan, following the Guide for the Use of Laboratory Animals (National Academy Press). EA pretreatment was applied by stainless steel acupuncture needles (1.5′′ inch, 30 G, Yu-kuang, Taiwan) that were inserted into the muscle layer at a depth of 2-3 mm at the ST36 acupoint. EA was performed after the injection of carrageenan or CFA and performed every day at the same time (12:00–14:00) for totally four days. Electrical square pulses were delivered for 15 min with duration of 1 ms and 2 Hz in frequency generated from the stimulator. The stimulation amplitude was 2 mA. The same treatment was given to nonacupoint (the gluteal muscle) to set as the sham control group [30].

### 2.2. Inflammatory Pain Models

Mice were anesthetized with 1-2% isoflurane and administered a single injection of 20 *μ*L saline (pH 7.4, buffered with 20 mM HEPES), 3% carrageenan (lambda carrageenan, type IV; Sigma), or CFA (complete Freund's adjuvant; 0.5 mg/mL heat-killed *M. tuberculosis* Sigma, St. Louis, MO) in the plantar surface of the hind paw to induce intraplantar inflammation [1, 31]. Behavior tests were conducted at day 4 after induction of inflammation, and DRGs were harvested on day 4 [30].

### 2.3. Animal Behavior of Mechanical, Thermal Hyperalgesia, and Hot/Cold Plate

Mechanical sensitivities were tested at 4 days after intraplantar injections. All experiments were performed at room temperature (approximately 25°C) and the stimuli were applied only when the animals were calm but not sleeping or grooming. Mechanical sensitivity was measured by testing the force of responses to stimulation with six applications of electronic von Frey filaments (North Coast Medical, Gilroy, CA, USA). Thermal pain was measured with six applications using Hargreaves' test IITC analgesiometer (IITC Life Sciences, SERIES8, Model 390G) [32]. Both hot/cold-induced pains were measured using a hot/cold plate (Panlab, Harvarf Apparatus). Five minutes of animal behavior were recorded using a digital camera and were analyzed offline using a personal computer [32, 33].

### 2.4. Immunohistochemistry and Image Analysis

Animals were anesthetized with an overdose of choral hydrate and intracardially perfused with saline followed by 4% paraformaldehyde. L3-L5 DRG neurons were immediately dissected and postfixed with 4% paraformaldehyde. Postfixed tissues were then placed in 30% sucrose overnight for cryoprotection. The DRGs were then embedded in OCT and rapidly frozen at −20°C. Frozen sections were cut in a 15 *μ*m thick on a cryostat. Samples were next incubated with blocking solution containing 3% BSA, 0.1% Triton X-100, and 0.02% sodium azide in PBS for 120 min at room temperature. After blocking, DRGs were incubated with primary antibodies prepared in blocking solution at 4°C overnight against TRPV1 (1 : 1000, Alomone) and TRPV4 (1 : 1000, Alomone). The secondary antibodies were goat anti-rabbit (Molecular Probes, Carlsbad, CA, USA). Slides were visualized by the use of fluorescence-conjugated secondary antibodies and mounted on cover slips. The images of TRPV1- and TRPV4-positive neurons were calculated to differentiate cell size using NIH ImageJ software (Bethesda, MD, USA) and showed the ratio of TRPV1- and TRPV4-positive staining in a different size.

### 2.5. Western Blot Analysis

L3-L5 DRG neurons were immediately excised to extract proteins. Total proteins were prepared by homogenized DRG in lysis buffer containing 50 mM Tris-HCl pH 7.4, 250 mM NaCl, 1% NP-40, 5 mM EDTA, 50 mM NaF, 1 mM Na_3_VO_4_, 0.02% NaN_3_, and 1× protease inhibitor cocktail (AMRESCO). The extracted proteins (30 *μ*g per sample assessed by a BCA protein assay) were subjected to 8% SDS-Tris glycine gel electrophoresis and transferred to a PVDF membrane. The membrane was blocked with 5% nonfat milk in TBS-T buffer (10 mM Tris pH 7.5, 100 mM NaCl, 0.1% Tween 20), incubated with anti-TRPV1 and TRPV4 antibody (1 : 1000, Alomone) in TBS-T with 1% bovine serum albumin, and incubated for 1 hour at room temperature. Peroxidase-conjugated anti-rabbit antibody (1 : 5000) was used as a secondary antibody. The bands were visualized by an enhanced chemiluminescent substrate kit (PIERCE) with LAS-3000 Fujifilm (Fuji Photo Film Co., Ltd.). Where applicable, the image intensities of specific bands were quantified with NIH ImageJ software (Bethesda, MD, USA).

### 2.6. DRG Primary Cultures and Whole-Cell Patch-Clamp Recording

CD1 mice aged 8–12 weeks were killed by use of CO_2_ to minimize their suffering. Lumbar (L3–L5) DRG neurons were dissected from ipsilateral site and placed in a tube containing DMEM and then transferred to DMEM with type I collagenase (0.125%, 120 min) for digestion at incubator at 37°C. Neurons were then plated on poly-L-lysine-coated cover slides. All recordings were completed within 24 hours after plating. Glass pipettes (Warner Products 64–0792) were prepared (2–5 MΩ) with the use of a vertical puller (NARISHIGE PC-10). Whole-cell recordings involved the use of an Axopatch MultiClamp 700B (Axon Instruments). Stimuli were controlled and digital records captured with the use of Signal 3.0 software and a CED1401 converter (Cambridge Electronic Design). Cells with a membrane potential more positive than −40 mV were not accepted. The bridge was balanced in current clamping recording and series resistance was compensated 70% in voltage-clamping recording with Axopatch 700B compensation circuitry. Recording cells were superfused in artificial cerebrospinal fluid (ACSF) containing (in mM) 130 NaCl, 5 KCl, 1 MgCl_2_, 2 CaCl_2_, 10 glucose, and 20 HEPES, adjusted to pH 7.4 with NaOH. ACSF solutions were applied by the use of gravity. The recording electrodes were filled with (in mM) 100 KCl, 2 Na2-ATP, 0.3 Na3-GTP, 10 EGTA, 5 MgCl_2_, and 40 HEPES, adjusted to pH 7.4 with KOH. Osmolarity was approximately 300–310 mOsm. Capsaicin was prepared from a 100-*μ*M stock solution (in 100% ethanol) to a final concentration of 1 *μ*M in ACSF. 4*α*-phorbol 12, 13-didecanoate (4*α*PDD) was prepared from a 300-*μ*M stock solution (in 100% ethanol) to a final concentration of 3 *μ*M in ACSF. All drugs were purchased from Sigma Chemical (St. Louis, MO, USA).

### 2.7. Statistical Analysis

All statistic data are presented as the mean ± standard error. Statistical significance between control, inflammation, and EA group was tested using the ANOVA test, followed by a post hoc Tukey's test (*P* < 0.05 was considered statistically significant).

## 3. Results

### 3.1. Low-Frequency EA at the ST36 Acupoint Decreased Carrageenan—and CFA-Induced Inflammatory Pain by the von Frey Filament and Hargreaves' Tests

**Figure 1:**
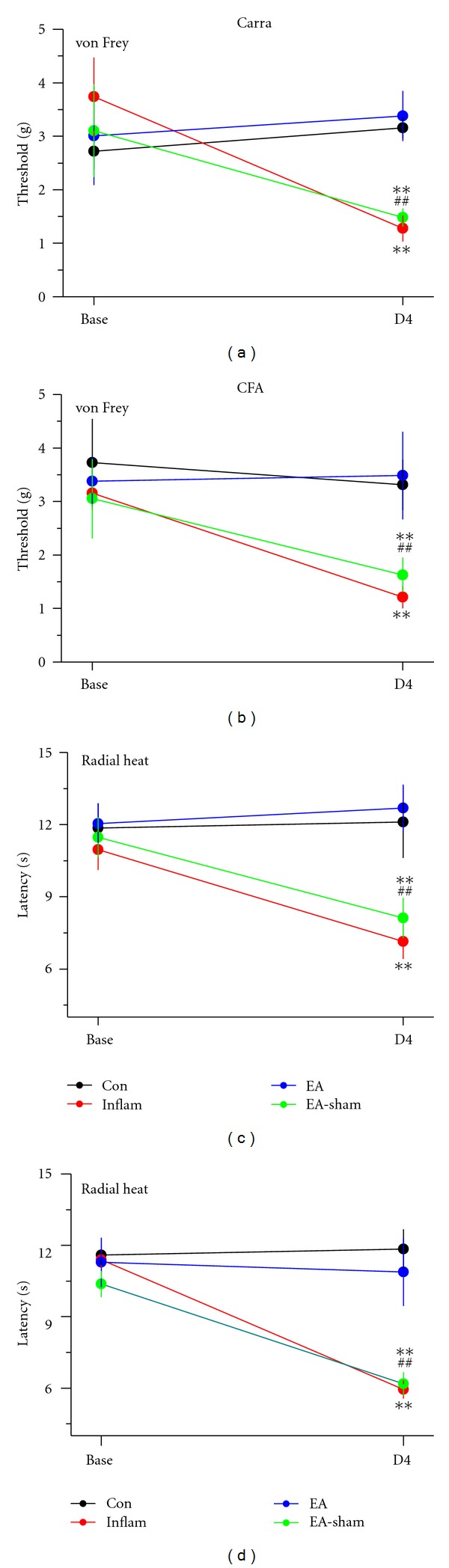
Acupuncture effectively decreased nociceptive responses in Carra (carrageenan) (a and c) and CFA- (complete-Freund's-adjuvant-) induced (b and d) inflammatory pain models. Mice from each inflammation model were tested for mechanical sensitivity with the electronic von Frey filament test (a and b) and thermal sensitivity with the radial heat test (c and d). The mice were tested before injection (Base) and on day 4 (D4). On day 4, mice were tested for inflammation-induced pain and the effect of EA (electroacupuncture) at ST36 and sham EA (EA-sham) was evaluated. For each model, four groups of mice were tested: Con (Control), Inflam (chemical-injected), EA (electroacupuncture), and EA-sham groups. **P < 0.01 compared with the baseline; ^##^ *P* < 0.01 for Inflam compared with EA-ST36 groups (*n* = 8 per group).

Intraplantar injection of normal saline did not initiate mechanical hyperalgesia (Figure 1(a), black circles: baseline, 2.72 ± 0.34 g; day 4, 3.15 ± 0.19 g; *n* = 8; *P* > 0.05). Intraplantar injection of carrageenan successfully evoked inflammation-mediated mechanical hyperalgesia (Figure 1(a), red circles: baseline, 3.74 ± 0.73 g; day 4, 1.28 ± 0.24 g; *n* = 8; *P* < 0.01). Low-frequency (2 Hz) EA at the ST36 acupoint reliably decreased carrageenan-induced inflammatory pain (Figure 1(a), blue circles: baseline, 3.01 ± 0.92 g; day 4, 3.38 ± 0.47 g; *n* = 8; *P* < 0.01). This decrease was not observed in the sham-EA group (Figure 1(a), green circles: baseline, 3.10 ± 0.86 g; day 4, 1.48 ± 0.17 g; *n* = 8; *P* > 0.05). These results suggested that EA at the ST36 acupoint had the potential to ameliorate inflammatory pain with acupoint specificity. We also examined CFA-induced inflammatory pain. The injection of CFA, but not normal saline, also induced mechanical hyperalgesia (Figure 1(b): control, black circles, 3.31 ± 0.47; CFA, red circles, 1.21 ± 0.21; *n* = 8 for each group). EA at the ST36 acupoint decreased CFA-mediated inflammatory pain, but sham EA did not (Figure 1(b): EA, blue circles, 3.48 ± 0.82; sham, green circles, 1.63 ± 0.32; *n* = 8 for each group). Thermal hyperalgesia was induced in carrageenan-injected mice but not in control mice (Figure 1(c): carrageenan, 7.14 ± 0.72 s; control, 12.1 ± 1.48 s; *n* = 8 for each group; *P* < 0.01). The carrageenan-induced thermal hyperalgesia could be attenuated by EA at the ST36 acupoint but not by sham EA (Figure 1(c): EA, blue circles, 12.61 ± 0.83 s; sham, green circles, 8.12 ± 0.38 s; *n* = 8 for each group; *P* < 0.01). Similar results were obtained with the CFA-induced thermal hyperalgesia (Figure 1(d)).

### 3.2. EA Attenuated Carrageenan-Elicited Inflammatory Pain by the Hot/Cold-Plate Pain Test

**Figure 2:**
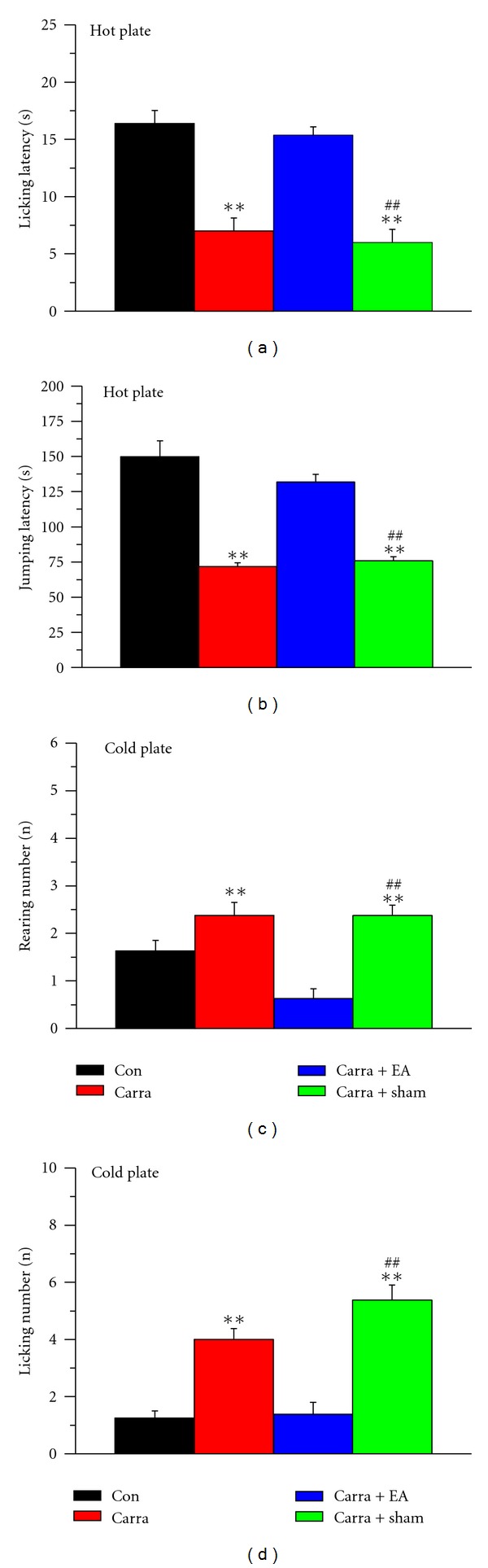
Acupuncture effects on nociceptive responses to noxious cold/hot plates after carrageenan induction. (a and b) Four groups of mice were exposed to a hot plate at 50°C, and licking and jumping latencies were analyzed. (c and d) The four groups were exposed to a cold plate at 4°C, and the rearing and licking numbers were analyzed. **P < 0.01 compared with the control group. ^##^ *P* < 0.01 for carra + sham compared with carra + EA groups (*n* = 8 per group). Con: control; carra: carrageenan-induced; EA: electroacupuncture at ST36; sham: EA at nonacupoint.

Intraplantar pretreatment with carrageenan significantly (*P* < 0.01) induced thermal hyperalgesia according to the licking latency parameter with a hot plate at 50°C at day 4 after injection (Figure 2(a): control, black, 16.38 ± 1.14 s; carrageenan, red, 7.0 ± 1.13 s; *n* = 8 for each group; *P* < 0.01). As shown in Figure 2(a), 2 Hz EA eliminated the effect of pain induced by carrageenan (blue, 15.38 ± 0.7 s), but sham EA did not (green, 6.0 ± 1.13 s; *n* = 8 for each group; *P* < 0.01). Furthermore, injection of carrageenan decreased the jumping latency induced with the hot plate at 50°C from 149.75 ± 11.38 s to 71.75 ± 2.74 s (Figure 2(b): black and red, respectively; *n* = 8 for each group; *P* < 0.01). The jumping latency was attenuated by 2 Hz EA but not by sham EA (Figure 2(b): blue and green, 131.88 ± 5.5 s and 75.88 ± 2.89 s, respectively; *n* = 8 for each group; *P* < 0.01), suggesting acupoint specificity. Next to verify the effect of carrageenan and EA on thermal hyperalgesia with a cold plate at 4°C, we analyzed rearing and licking numbers in the four groups. We consistently found that intraplantar injection of carrageenan significantly increased the rearing number from 1.63 ± 0.22 to 2.38 ± 0.28 (Figure 2(c): black and red, respectively; *n* = 8 per group; *P* < 0.01). Interestingly, 2 Hz EA also had a potential effect on cold hyperalgesia induced by carrageenan injection compared with sham EA-treated mice (Figure 2(c): EA, blue, 0.63 ± 0.21; sham, green, 2.38 ± 0.22; *n* = 8 per group; *P* < 0.01). Injection of carrageenan dramatically increased the licking number from 1.25 ± 0.25 to 4.0 ± 0.38, suggesting thermal hyperalgesia with assessment with the cold plate at 4°C (Figure 2(d): black and red, respectively; *n* = 8 per group; *P* < 0.01). EA cured the carrageenan-induced hyperalgesia estimated with the cold plate, but sham EA did not (Figure 2(d): blue and green, 1.38 ± 0.42 and 5.38 ± 0.53, resp.; *n* = 8 per group; *P* < 0.01).

### 3.3. EA Attenuated CFA-Elicited Inflammatory Pain by the Hot/Cold-Plate Pain Test

**Figure 3:**
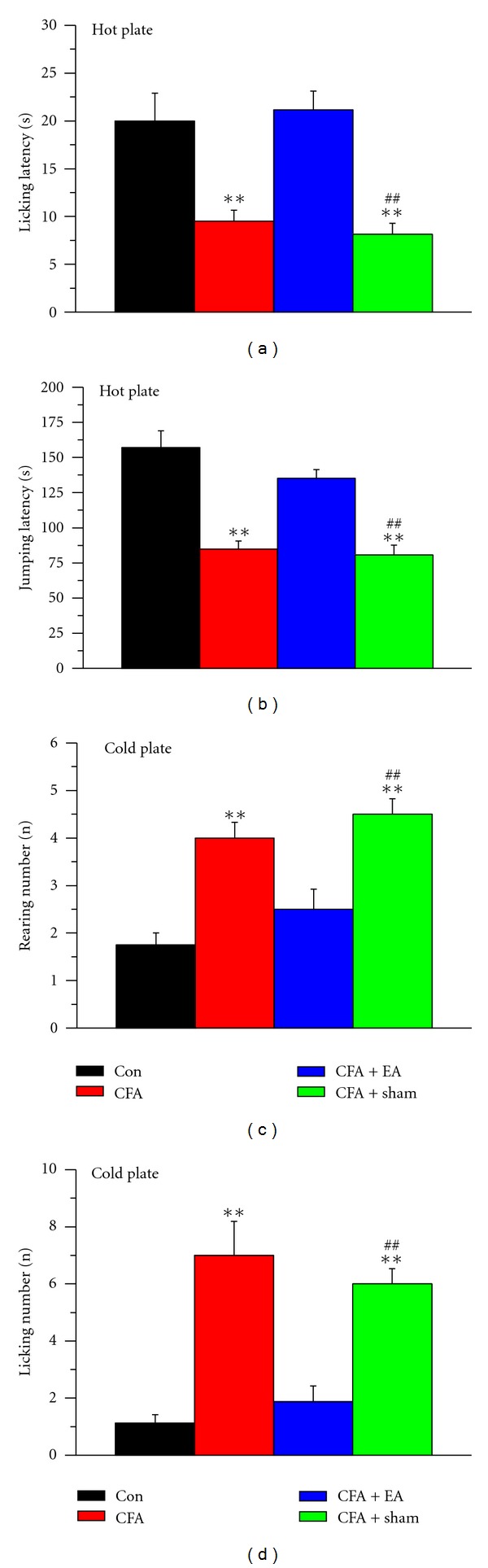
Acupuncture effects on nociceptive responses to noxious cold/hot plates after CFA (complete Freund's adjuvant) induction. (a and b) Four groups were exposed to a hot plate at 50°C, and the licking and jumping latencies were analyzed. (c and d) The four groups were exposed to a cold plate at 4°C cold, and the rearing and licking numbers were analyzed. **P < 0.01 compared with the control group. ^##^ *P* < 0.01 for CFA + sham compared with CFA + EA groups (*n* = 8 per group). Con: control; EA: electroacupuncture at ST36; sham: EA at nonacupoint.

Intraplantar injection of CFA consistently induced thermal hyperalgesia according to the licking latency parameter (Figure 3(a): control, black, 20.1 ± 2.89 s; CFA, red, 9.5 ± 1.17 s; *n* = 8 per group; *P* < 0.01). EA at the ST36 acupoint decreased this hyperalgesia, but sham EA did not (Figure 3(b): EA, blue, 21.13 ± 1.99 s; sham EA, green, 8.13 ± 1.17 s; *n* = 8 per group; *P* < 0.01). CFA injection decreased the jumping latency with the hot plate at 50°C from 157.0 ± 11.94 s to 84.75 ± 5.67 s (Figure 3(b): control, black; CFA, red; *n* = 8 per group; *P* < 0.01). The jumping latency was increased by EA stimulation but not by sham EA (Figure 3(b): EA, blue, 135.13 ± 6.25 s; sham EA, green, 80.75 ± 6.90 s; *n* = 8 per group; *P* < 0.01). We also characterized the cold-mediated hyperalgesia in each group. CFA injection increased the rearing number from 1.75 ± 0.25 to 4.0 ± 0.33 (Figure 3(c): black and red, respectively; *n* = 8 for each group; *P* < 0.01). EA decreased rearing induced by CFA injection, but sham EA did not (Figure 3(c): EA, blue, 2.5 ± 0.42; sham, green, 4.5 ± 0.33; *n* = 8 per group; *P* < 0.01). CFA injection increased the licking number from 1.13 ± 0.29 to 7.0 ± 1.19 with the cold-plate assessment (Figure 3(d): black and red, respectively; *n* = 8; *P* < 0.01). EA could reverse the CFA-induced hyperalgesia evaluated with the cold plate, but sham EA did not (Figure 3(d): EA, blue, 1.88 ± 0.55; sham EA, green, 6.0 ± 0.53; *n* = 8 per group; *P* < 0.01).

### 3.4. EA at the ST36 Acupoint Altered Electrophysiological Properties in Inflamed DRG Neurons

**Figure 4:**
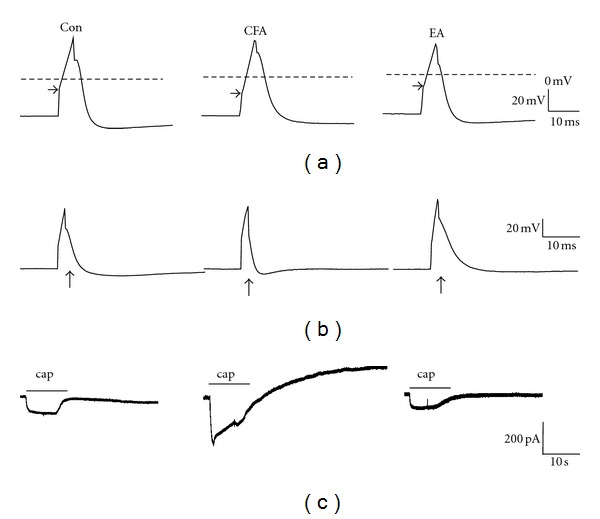
Electrophysiological properties of AP (action potential) and TRPV1-mediated inward currents in the Con (control), CFA (complete Freund's adjuvant), and EA (electroacupuncture) groups. (a) The AP threshold was more negative in the CFA group than in the control group. EA attenuated neuron excitability by reversing the AP threshold to a positive membrane potential. (b) CFA-induced inflammation decreased both the rising and falling times compared with the control group. EA treatment decreased the neuron excitability to the control level. (c) The capsaicin- (cap-) induced inward current was increased in CFA-treated DRG neurons compared with the control group. This was reversed by EA manipulation.

 We examined the membrane properties of acutely isolated DRG neurons through whole-cell patch clamp recordings. Compared with the control group, DRG neuronal excitability was increased in mice 4 days after CFA-induced inflammation. The resting membrane potential and capacitance were similar among the control, CFA-induced, and EA-treated groups, indicating similar properties of the neurons. The AP threshold and rheobase were decreased in the CFA-inflamed group, indicating increased excitability, and these were attenuated in the EA group (Figure 4(a)). In addition, both the AP rise and fall times were significantly shorter for neurons in the inflammation group compared with those in the control group, and this result was reversed in the EA-treated group (Figure 4(b)). Moreover, no significant differences were found among the three groups in AP amplitude and after hyperpolarization (AHP) duration. To investigate the electrophysiological properties of TRPV1 and TPRV4, we injected TRPV1 or TPRV4 specific agonist capsaicin or 4*α*PDD to primary cultured DRG neurons to induce inward current. Notably, the percentage of TRPV1-positive neurons and the amplitude of TRPV1-induced inward current induced by the TRPV1 agonist capsaicin were potentiated by CFA-elicited inflammation and further ameliorated by EA treatment (Figure 4(c)). Similar results were also observed in TRPV4 agonist 4*α*PDD induced neurons. The statistically analyzed data are presented in Table 1.

### 3.5. TRPV1 and TRPV4 Expression in DRG Neurons from Carrageenan-Induced Hyperalgesia Was Decreased by EA

**Figure 5:**
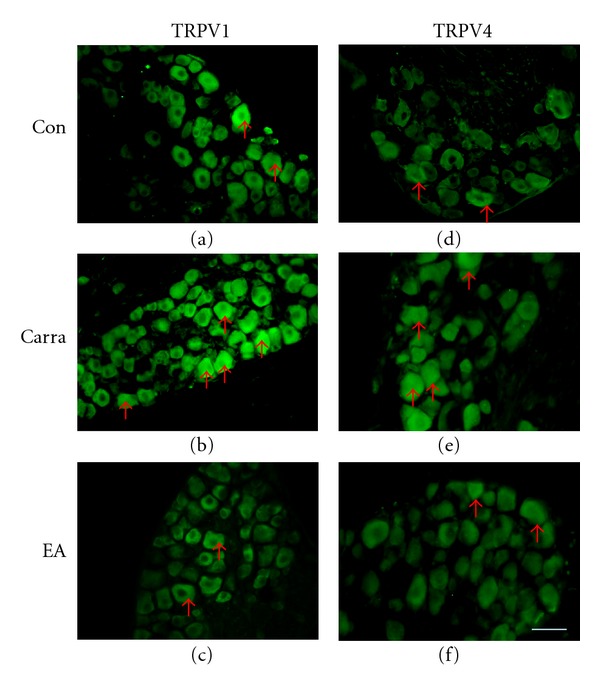
TRPV1 and TRPV4 expression was increased in DRG (dorsal root ganglia) after intraplantar carrageenan injection and attenuated by electroacupuncture (EA) at the ST36 acupoint in mice. (a–c) Mouse DRGs were stained with TRPV1 antibody. (d–e) Mouse DRGs were stained with TRPV4 antibody. Arrows indicate positive antibody reactions. Scale bar = 50 *μ*m for all panels. Con: control; Carra: carrageenan-induced; EA: electroacupuncture at ST36.

To correlate the development of inflammatory pain and the curative effects of EA with changes in TRPV1 and TRPV4 in DRG neurons, we first used immunohistochemistry to verify TRPV1 and TRPV4 expression. The expression of TRPV1 was observed in DRG neurons (Figure 5(a)). Following intraplantar injection of carrageenan, the TRPV1 staining intensity significantly increased in DRG neurons (Figure 5(b)). This increased expression of TRPV1 reverted to that of the normal control group with 2 Hz EA (Figure 5(c)). TRPV4 was also present in DRG neurons (Figure 5(d)) and its expression increased after carrageenan injection (Figure 5(e)). The overexpression of TRPV4 was attenuated by EA stimulation (Figure 5(f)).

### 3.6. EA Abated the CFA-Mediated Inflammatory Pain Response by Altering TRPV1 and TRPV4 Levels

**Figure 6:**
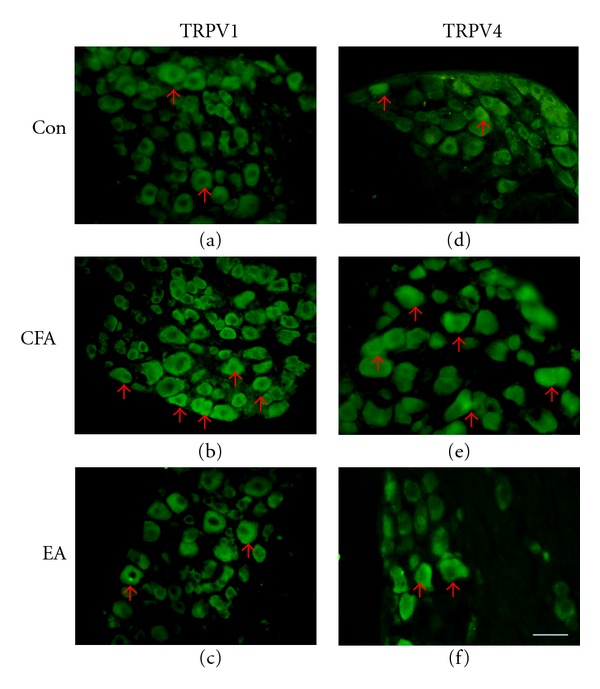
TRPV1 and TRPV4 expression was increased in DRG (dorsal root ganglia) after intraplantar CFA (complete Freund's adjuvant) injection and attenuated by EA (electroacupuncture) at the ST36 acupoint in mice. (a–c) Mouse DRGs were stained with TRPV1 antibody. (d–e) Mouse DRGs were stained with TRPV4 antibody. Arrows indicate positive antibody reactions. Scale bar = 50 *μ*m for all panels. Con: control. CFA: CFA-induced; EA: electroacupuncture at ST36.

**Figure 7:**
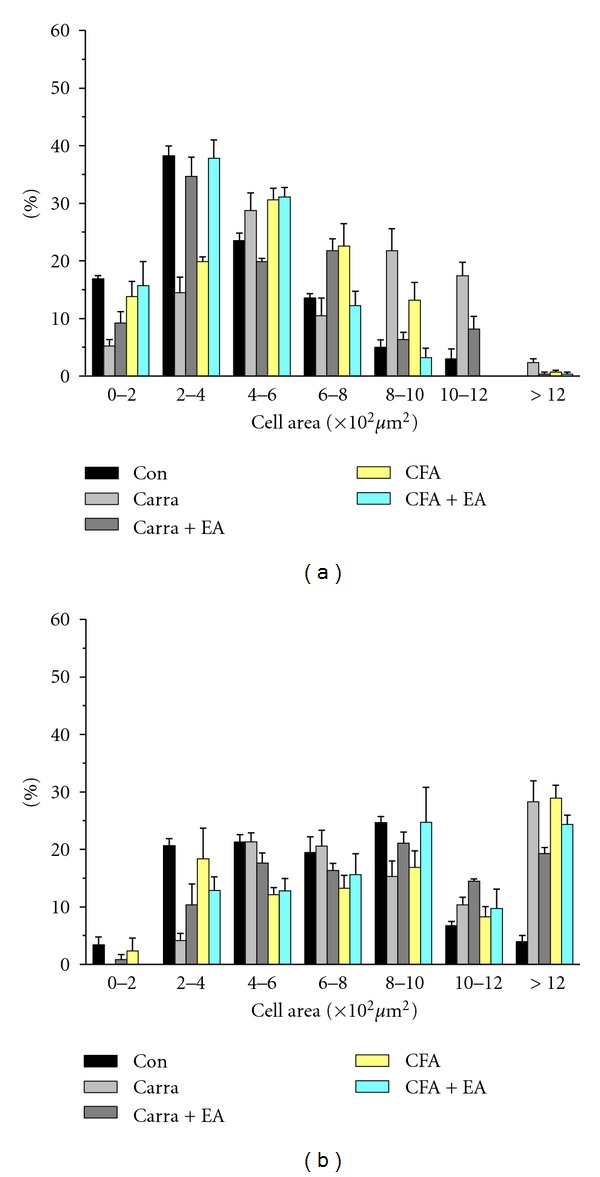
Cell area percentage of TRPV1-immunoreactive neurons in the L3-L5 DRG in mice in control, carrageenan, carrageenan with EA (electroacupuncture), CFA (complete Freund's adjuvant), and CFA with EA (electroacupuncture) treatment. (a) The percentage of TRPV1-positive neurons from lumbar DRGs that belonged to corresponding cell area was presented. At day 4, TRPV1-positive neurons were dominantly observed in small neurons (cell area < 800 *μ*m^2^). The percentage of TRPV1-positive neurons were significantly increased with carrageenan and CFA treatment and further reduced with EA manipulation (*P* < 0.05). (b) The TRPV4-positive neurons were majorly found in medium to large diameter neurons and did not significantly alter in inflammatory pain model (*P* > 0.05).

We next determined the alterations in TRPV1 and TRPV4 levels in CFA-elicited inflammatory hyperalgesia. TRPV1 levels were normal in DRG neurons (Figure 6(a)) and increased following CFA injection (Figure 6(b)). This phenomenon was reversed by 2-Hz EA stimulation at the ST36 acupoint (Figure 6(c)). TRPV4 also was observed in DRG neurons (Figure 6(d)). TRPV4 levels increased following CFA injection (Figure 6(e)) and were dramatically attenuated by 2-Hz EA stimulation (Figure 6(f)). Cell area versus frequency histograms (Figure 7) showed that TRPV1 proteins were mainly presented in small neurons (cell area < 800 *μ*m^2^). At day 4 after carrageenan injection, the TRPV1-reactive neurons were increased within small-medium (800–1200 *μ*m^2^) neurons compared with control group (*P* < 0.05). The potentiation of TRPV1 protein level was attenuated with EA stimulation. The similar results were obtained in a CFA-treated group. The TRPV4-positive neurons were mainly expressed in medium to large neurons and the ratio of all the population did not alter with both carrageenan and CFA-induced inflammatory pain models. However, our Western blot found TRPV4 was increased with both carrageenan and CFA-elicited inflammatory pain models; we suggested that TRPV4 was increased in all types of DRGs.

### 3.7. EA at ST36 Ameliorated Overexpression of TRPV1 and TRPV4 in DRG Neurons by Western Blotting

**Figure 8:**
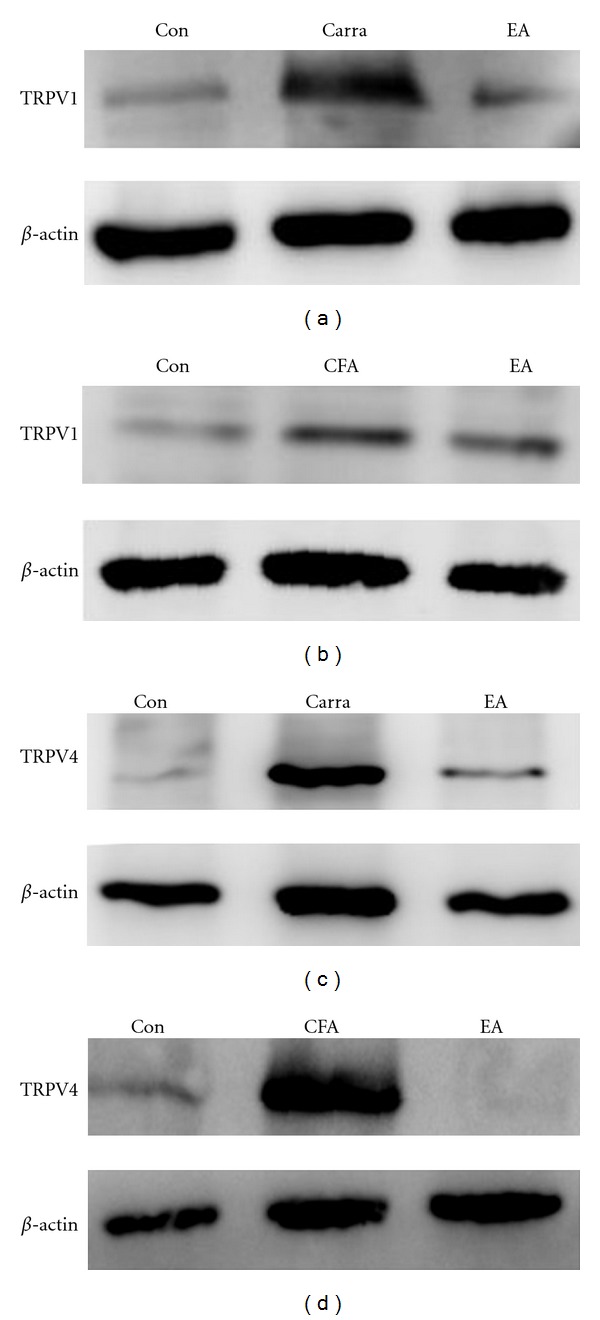
TRPV1 and TRPV4 protein levels. DRG (dorsal root ganglion) lysates underwent immunoreactions with specific TRPV1 (a and b) and TRPV4 (c and d) antibodies. TRPV1 and TRPV4 increased substantially after carra (carrageenan) or CFA (complete Freund's adjuvant) injection as compared with the saline-injected group (Con). TRPV1 and TRPV4 protein levels were attenuated by electroacupuncture (EA) at the ST36 acupoint as compared with the carra- and CFA-induced groups.

We used Western blotting to further analyze the levels of TRPV1 and TRPV4 proteins in DRG neurons. TRPV1 protein was expressed normally in the control group. After carrageenan-induced hyperalgesia, TRPV1 protein expression was greatly increased (Figure 8(a), 141.37 ± 7.59% compared with control group; *n* = 6, *P* < 0.05). This increase was effectively downregulated by 2 Hz EA stimulation at the ST36 acupoint (Figure 8(a), 83.25 ± 13.34% compared with carrageenan group, *n* = 6; *P* < 0.05). TRPV4 protein was also overexpressed after carrageenan injection (Figure 8(c), 214.66 ± 11.25% compared with control group, *n* = 6; *P* < 0.05) and attenuated by 2 Hz EA stimulation at the ST36 acupoint (Figure 8(c), 113.07 ± 4.22% compared with carrageenan group, *n* = 6; *P* < 0.05). Similar phenomena were observed with CFA-induced inflammation (Figures 8(b) and 8(d)). These results demonstrated that 2 Hz EA at the ST36 acupoint could successfully reverse inflammation-induced TRPV1 and TRPV4 overexpression in both the carrageenan- and CFA-induced models.

## 4. Discussion 

EA at ST36 can effectively decrease inflammation-induced pain, but the detailed mechanism remains unknown [34, 35]. Both TRPV1 and TRPV4 are highly correlated with mechanical and thermal pain. We hypothesized that EA at ST36 could attenuate inflammation-induced pain through the mediation of TRPV1 and TRPV4 channels.

TRPV1 and TRPV4 are both cation channels that are activated at temperatures over 43°C or 25°C, and both are essential for thermal and mechanical hyperalgesia [22, 36]. Both are highly expressed in DRG neurons after inflammation induction that results in thermal and mechanical hyperalgesia [22, 36]. Many reports have found that TRPV1 and TRPV4 antisense oligonucleotides or antagonists can effectively ameliorate thermal and mechanical hyperalgesia. Moreover, depletion of TRPV1 or TPRV4 in mice results in higher withdrawal latencies in the von Frey's or Hargraves' tests. These data indicate that both TRPV1 and TRPV4 are essential for mediating thermal and mechanical sensations [22, 23, 36]. Our results show that thermal and mechanical sensitivities, as measured by hot-plate-induced licking and jumping latency, are altered following inflammatory pain and that these phenomena are attenuated by 2 Hz EA stimulation. We suggest that these behavioral changes are mediated through TRPV1 and TRPV4 downregulation by a 2 Hz EA. Our data also show that a cold plate at 4°C increases rearing and licking numbers in mice and that this result is abated by 2 Hz EA. These results suggest that EA may also regulate different channels such as TRPA1 or TRPM8 [23].

It has long been recognized that TRPV1 is involved in pain sensations and can increase synaptic transmission in the hippocampus, hypothalamus, and spinal cord with increased miniature excitatory postsynaptic current (mEPSC) frequency after capsaicin application [37, 38]. Activation of TRPV1 induces long-term depression in the nucleus accumbens and hippocampal dentate gyrus [37, 38]. TRPV1 is suggested to mediate both thermal and mechanical hyperalgesia in inflammatory hyperalgesia. Deletion of TRPV1 is crucial for decreasing CFA-elicited mechanical and thermal hyperalgesia in knee joint and muscle inflammation models. TRPV1 antagonists or antisense oligonucleotides have similar effects in decreasing inflammatory pain symptoms [10, 36]. TRPV1 is reported to be activated by mediators and secondary messengers in inflammatory conditions and with tissue injury and ischemia. Under these conditions, peripheral acidosis with low pH is also thought to activate TRPV1 and contribute to pain sensation [23]. Our results suggest that both carrageenan and CFA injection can enhance TRPV1 protein levels in peripheral DRGs. Furthermore, EA manipulation can reliably ameliorate this inflammation-induced upregulation of TRPV1. This phenomenon has also been observed in a tumor pain model, and the powerful therapeutic effect of TRPV1 blockage is being explored [34]. 

Peripheral synaptic transmission from DRG neurons to the spinal cord dorsal horn (SCDH) is crucial for pain signaling [3]. At these synapses, glutamate is released from presynaptic nerve terminals by several types of stimuli and binds to postsynaptic receptors. These signals are further transferred into electrical signals to the brain for pain sensation and pain responses [39]. In the inflammatory pain process, the probability of augmented glutamate release leads to central nervous system sensitization. Our results show that carrageenan- and CFA-induced inflammatory pain reliably induces mechanical and thermal pain accompanied by TRPV4 increase, as shown by immunostaining and Western blotting. This phenomenon can be reversed through low-frequency (2-Hz) EA stimulation. This is a novel mechanism underlying acupuncture therapy. Recently, activation of TRPV4 by application of its agonist, 4*α*PDD, was shown to significantly potentiate the frequency of mEPSCs, implying that presynaptic transmission is responsible for TRPV4 action [38]. Cao and colleagues have demonstrated that TRPV4-elicted membrane currents and synaptic transmission occur primarily through protein kinase C activation [38]. Accordingly, increased TRPV4 may result in enhanced excitability of pain signaling and further induce central sensitization. Mechanical hyperalgesia is decreased in animals by intrathecal administration of TRPV4 antisense oligonucleotides or TRPV4 gene depletion [19, 40]. Ding et al. also have reported that TRPV4 is crucial for the thermal pain process induced by chronic compression of DRG neurons in rats through mechanisms that activate TRPV4-NO-cGMP-PKG pathways [41]. 

Recent studies have shown that ATP is released at ST36 after acupuncture, and ATP is metabolized to adenosine by specific enzymes [42]. Activation of the A1R by adenosine decreases TPRV1 activation by depleting PIP2 (phosphatidylinositol 4, 5-bisphosphate), because PIP2 is important for TRPV1 channel activation [43]. Our results show that TRPV1 expression and physiological function are affected by acupuncture, and we suggest that this phenomenon is influenced by A1R activation. Chen et al. have also reported that it activates PAR2 (protease-activated receptor 2), which then activates PKA (protein kinase A) and PKC (protein kinase C), causing mechanical and thermal (both heat and cold) hypersensitivity. Furthermore, this hypersensitivity is effectively inhibited by TRPV1 and TRPV4 antagonists [44]. A1R is a GPCR (G-protein-coupled receptor), and activation of A1R decreases adenylyl cyclase activity through activation of pertussis toxin-sensitive Gi proteins that then inhibit PKA activity [45–47]. We suggest that the mechanism underlying acupuncture-mediated analgesia may be A1R activation, which then inhibits PKA activation resulting in downregulation of TRPV1 and TRPV4.

Studies of the mechanism of pain signaling may lead to the development of additional drugs and therapies. Hurt and colleagues have reported that PAP (prostatic acid phosphatase) is an ectonucleotidase that can hydrolyze extracellular AMP to adenosine in the nociceptive system. Injection of PAP to the Weizhong acupoint has antinociceptive effects in mouse inflammatory pain models [48]. Therese and colleagues have found that TRPV1 is more highly expressed at the BL40 acupoint skin than in the nonacupoint control skin and that TRPV1 expression can be influenced by EA stimulation [49]. This indicates that TRPV1 is associated with the BL40 acupoint. Our data show that acupuncture can mediate TPRV1 and TRPV4 expression in DRG neurons. Furthermore, we may be able to develop more effective therapies by combining a TPRV1 antagonist or agonist with acupoints to prolong the effects of acupuncture therapy.

## 5. Conclusion

This current study suggests that TRPV1 and TRPV4 were augmented in mice DRG neurons in both the carrageenan and CFA-induced inflammatory pain models. Accordingly, this is the first paper regarding the functional role of acupuncture in pain signaling and its novel findings regarding TRPV1 and TRPV4 channel downregulation. The phenomenon can be further attenuated by EA at the ST36 acupoint, rather than sham group. These results indicate highly valuable data from investigating acupuncture-mediated analgesia mechanisms and can be further applied to clinical medicine.

## Figures and Tables

**Table 1:** Electrophysiological analysis of action potential parameter, TRPV1, and TRPV4 current properties.

	Con (*n* = 39)	CFA (*n* = 30)	EA (*n* = 28)
Resting membrane potential (mV)	−46.70 ± 1.72	−44.60 ± 1.42	−42.04 ± 2.41
Capacitance (pF)	35.39 ± 1.83	37.29 ± 1.84	37.93 ± 4.29
AP threshold (mV)	−19.93 ± 1.35	−25.04 ± 2.58**	−17.66 ± 2.86^#^
AP rheobase (pA)	483.03 ± 41.80	401.83 ± 22.92*	600.10 ± 58.79^##^
AP amplitude (mV)	78.35 ± 2.28	73.82 ± 3.31	74.79 ± 4.74
AP rise time (ms)	2.30 ± 0.11	2.07 ± 0.02*	2.49 ± 0.14^##^
AP fall time (ms)	6.36 ± 0.37	3.96 ± 0.39**	8.41 ± 2.28^#^
AHP (ms)	71.69 ± 8.24	60.22 ± 9.43	77.14 ± 10.14
TRPV1%	20.51	30	14.29
TRPV4%	17.95	33.33	17.85
TRPV1 amplitude (pA)	90.79 ± 12.99	189.53 ± 39.38*	67.74 ± 11.53^#^
TRPV4 amplitude (pA)	99.35 ± 14.54	374.24 ± 108.59*	129.48 ± 24.93^#^

*P < 0.05 compared with the control group; **P < 0.01 compared with the control group; ^#^ *P* < 0.05 between the inflammation and EA groups; ^##^ *P* < 0.01 between the inflammation and EA groups. Con: control; CFA: complete Freund's adjuvant; EA: electroacupuncture; AP: action potential; AHP: afterhyperpolarization.
